# A Deep Learning Based Platform for Remote Sensing Images Change Detection Integrating Crowdsourcing and Active Learning

**DOI:** 10.3390/s24051509

**Published:** 2024-02-26

**Authors:** Zhibao Wang, Jie Zhang, Lu Bai, Huan Chang, Yuanlin Chen, Ying Zhang, Jinhua Tao

**Affiliations:** 1 School of Computer and Information Technology, Northeast Petroleum University, Daqing 163318, China; wangzhibao@nepu.edu.cn (Z.W.); zhangjie@stu.nepu.edu.cn (J.Z.); 228003071186@stu.nepu.edu.cn (H.C.); chenyuanlin@aircas.ac.cn (Y.C.); 2 Bohai-Rim Energy Research Institute, Northeast Petroleum University, Qinhuangdao 066004, China; 3 School of Electronics, Electrical Engineering and Computer Science, Queen’s University Belfast, Belfast BT9 6SB, UK; 4 State Key Laboratory of Remote Sensing Science, Aerospace Information Research Institute, Chinese Academy of Sciences, Beijing Normal University, Beijing 100101, China; zhangying01@radi.ac.cn (Y.Z.); taojh@radi.ac.cn (J.T.)

**Keywords:** remote sensing, change detection, crowdsourcing, active learning, human-in-the-loop

## Abstract

Remote sensing images change detection technology has become a popular tool for monitoring the change type, area, and distribution of land cover, including cultivated land, forest land, photovoltaic, roads, and buildings. However, traditional methods which rely on pre-annotation and on-site verification are time-consuming and challenging to meet timeliness requirements. With the emergence of artificial intelligence, this paper proposes an automatic change detection model and a crowdsourcing collaborative framework. The framework uses human-in-the-loop technology and an active learning approach to transform the manual interpretation method into a human-machine collaborative intelligent interpretation method. This low-cost and high-efficiency framework aims to solve the problem of weak model generalization caused by the lack of annotated data in change detection. The proposed framework can effectively incorporate expert domain knowledge and reduce the cost of data annotation while improving model performance. To ensure data quality, a crowdsourcing quality control model is constructed to evaluate the annotation qualification of the annotators and check their annotation results. Furthermore, a prototype of automatic detection and crowdsourcing collaborative annotation management platform is developed, which integrates annotation, crowdsourcing quality control, and change detection applications. The proposed framework and platform can help natural resource departments monitor land cover changes efficiently and effectively.

## 1. Introduction

With the advancement of earth observation technology, the number of global remote sensing satellites with land cover capability has increased year after year [1]. The satellite’s broad coverage and high timeliness monitoring capabilities have attracted more attention for its application in remote sensing image change detection. Using remote sensing images to detect land cover changes has an essential value in land use, land cover monitoring, and natural disaster assessment [2,3,4,5,6,7]. Automated and intelligent remote sensing change detection technology monitors natural disasters such as earthquakes, floods, and volcanic eruptions. It enables early detection and early control, reduces the losses caused by disasters, and enables the detection of recent land cover changes caused by human violations [8,9]. In recent years, with the rapid development of urbanization, many forest land reserves have been deforested, illegally constructed, and rebuilt, turning more cultivated land into non-agricultural land. The traditional land cover change detection is performed through manual visual interpretation with a low degree of automation and an extensive manual workload, and suffers from problems such as low efficiency, high missed detection rates, and poor timeliness. Therefore, there is a growing need for real-time intelligent change detection.

With the recent advancement of the integration of artificial intelligence technology with remote sensing image interpretation technology, scholars have successively applied deep learning technology to the field of remote sensing image analysis. Compared with the traditional machine learning methods by manually extracting features, the deep learning methods can automatically learn the complex change characteristics of remote sensing images and adapt to the problem of diverse scenes in change detection from remote sensing images. Recently, several pieces of research have been conducted in remote sensing image target extraction, change detection, and land cover classification using deep learning-based methods [10,11,12,13]. The quality and quantity of data samples are the key factors in improving the performance of the remote sensing change detection model. Many optical remote sensing images change detection datasets have been published and provided open access to users, such as the BCDD dataset [14], the OSCD dataset [15], and the HRSCD dataset [16]. These remote sensing open datasets provide data for remote sensing change detection research. However, most of the remote sensing datasets are not labeled. Labeling is time-consuming and costly for large-scale remote sensing datasets and requires professional domain knowledge. To address challenges in the annotation, it is possible to use crowdsourcing technology [17] by assigning the task of massive remote sensing image semantic annotation to people from online public groups. The crowdsourcing technology is dependent on the quantity and quality of labeled samples. For specific change detection scenarios, human-in-the-loop technology [18] can be used as a feasible solution to the problem of poor detection results when the existing model is applied to the new dataset. Moreover, to allow the model to adapt to the changing environment quickly, the active learning method [19,20] is used to find out the uncertain samples through the query strategy, and then use the crowdsourcing platform to annotate these uncertain samples. The main aim is to integrate human experience into model learning so that the models can rely on human intelligence to become more intelligent.

Change detection remains one of the most important tasks in remote sensing analysis, and it has aroused worldwide interest in recent years with the explosive development of artificial intelligence [21]. Change detection enables the dynamic monitoring of different types of images acquired through remote sensing satellite sensors. Xu et al. [22] adopted a cross-attention module to enhance the capability of extracting and characterizing the features of the changed region, reducing the influence of noise and pseudo-change of high-resolution cropland resource images. Eismann et al. [23] proposed algorithms to detect changes in the presence of diurnal and seasonal variations for hyperspectral remote sensing. Zhang et al. [24] proposed a U-Net + LSTM framework to detect the changes in deforestation from optical remote sensing images.

Most of the current literature is based on theoretical research on the combination of active learning and deep learning. The practical application of deep learning in remote sensing image change detection and active learning is relatively limited. The remote sensing change detection technology based on deep learning has been widely applied to land cover change monitoring, urban development change analysis, and natural disaster assessment. However, there is still a gap in achieving efficient automation and obtaining good change detection accuracy. This is mainly due to the lack of large-scale, high-quality annotated change detection datasets. Therefore, this paper proposed a crowdsourcing collaborative annotation platform integrated with the active learning method and human-in-the-loop technique. It aims to achieve automatic change detection for remote sensing images with solid versatility and comprehensive application scenarios.

The main contribution of this work is as follows:

- A platform for large-scale change detection of remote sensing images is developed with the integration of crowdsourcing, human-in-the-loop, and active learning techniques;
- A quality control model for crowdsourcing is proposed, combined with the annotator ability assessment model;
- An active learning approach is proposed utilizing annotated data from crowdsourcing in remote sensing images change detection.

## 2. Related Works

### 2.1. Deep Learning-Based Change Detection Models

Monitoring land use and land cover from satellite images is critical in earth observation [25]. Identifying areas of transformation across multiple images of the same scene captured in various instances attracts significant attention, driven by its multitude of applications across diverse disciplines, such as in remote sensing [26,27,28]. Many methods have been used for change detection from satellite images. The algebra-based change detection method [29] mainly obtains the image difference by comparing each pixel using mathematical operations. Classification-based methods include post-classification comparison and unsupervised change detection methods [25,30]. Deep learning-based change detection methods have succeeded in many remote sensing change detection studies. An object-based land-use and land-cover change detection was performed for a case study in Ardabil, Namin, and Nir counties in Northwest Iran [31]. For unsupervised change detection methods, Kusetogullari et al. [32] proposed an unsupervised algorithm for change detection in multitemporal multispectral satellite images using parallel particle swarm optimization. Cao et al. [33] proposed a new approach for unsupervised change detection in high-resolution remote sensing images utilizing a Conditional Random Field (CRF) model. Change detection techniques can be applied to various types of satellite images, including Synthetic Aperture Radar [34] and multispectral images [35]. The Long Short-Term Memory (LSTM) model has been widely used to detect land use changes from remote sensing images [36,37]. U-shape neural network architecture (U-Net) [38] was initially used for biomedical image segmentation but was then widely used for remote sensing change detection [39,40,41]. U-Net++ [42] is an improved version of U-Net, which provides a better solution for change detection of high-resolution satellite images [41,43]. Chaurasia and Culurciello proposed LinkNet [44], an efficient pixel-wise semantic segmentation method. Modified LinkNet has been successfully used in satellite imagery road extraction [45] and change detection [46]. PSPNet [47] provides a superior framework for pixel-level prediction, and many change detection applications adopt this method [48]. Feature pyramid network (FPN) [49] has been widely used in generating feature representations at the feature extraction stage. Ref. [50] used FPN to generate features for the change detection model. DeepLabV3plus [51] has been used as a semantic segmentation model, and it has been used in several change detection tasks recently [52]. 

### 2.2. Crowdsourced Annotations

In computer vision, remote sensing image-annotated datasets are far less than natural image-annotated datasets. Labeling remote-sensing images takes more time and requires domain knowledge and experience in remote sensing [53]. Crowdsourcing technology provides a good solution for tasks that require both human intelligence and machine intelligence, which can be a feasible option for labeling large-scale remote sensing image data [54]. Ref. [55] researched crowdsourced image annotation for pathology imaging experiment results and assigned the annotation task to annotators according to the crowdsourcing task’s complexity and the annotators’ professional level. Multiple crowdsourced annotation results were gathered for each image and further used to improve annotation quality. In remote sensing, the Geo-Wiki project [56] allows volunteers worldwide to collaborate to help produce more accurate land cover maps. OpenStreetMap (OSM) [57] has a large and prosperous vector dataset provided by volunteers around the world and is often used to train machine learning models [58]. In order to solve the problem of insufficient training data in remote sensing image classification, Saralioglu and Gungor (2022) developed a crowdsourcing platform to label image data. They used the CNN network to verify the accuracy of the labeled data, with an average accuracy rate of more than 95%. It can be seen from existing research that crowdsourcing annotation technology is playing an increasingly important role in the field of remote sensing image data annotation.

Since annotation integrates unknown people on the Internet to work, there can be fluctuations in annotation quality during the annotation process, which may result in uncertainty in the quality of submitted annotation results. Therefore, the problem of annotation quality detection is also a current challenge in this area. An annotation quality control algorithm is required to ensure the quality of annotation. The majority voting algorithm (MV) [59,60,61,62] has been widely used to determine the final labeling result, where the same labeling task is assigned to multiple crowd workers to complete. However, the MV algorithm assumes that each crowd worker has the same labeling qualifications, and the differences in the labeling ability of the crowdsourcing workers are not considered. The crowd worker can widely vary in skills of labeling results. In response to the above problem, some researchers estimate the accuracy of the annotation results used to evaluate the annotation qualifications of the crowd workers, which significantly improved the accuracy of the final annotation results. Ref. [63] calculated the labeling accuracy of the crowdsourced workers by adding the gold standard data test task and used the Bayesian algorithm to calculate the final quality result by combining the labeling accuracy of the crowdsourced workers and the labeling results. Ref. [64] proposed a method to represent the variation of the annotation accuracy of crowdsourcing workers through a confusion matrix based on the expectation maximization (EM) algorithm [65]. Ref. [66] evaluated a variety of crowdsourced quality control algorithms for crowdsourced image semantic annotation quality. Ref. [67] proposed a general crowd worker annotation quality evaluation algorithm suitable for all annotation tasks, such as image classification, text classification, speech recognition, and video classification. Ref. [68] experimented with evaluating the annotation qualifications of crowd workers on the MTurk crowdsourcing platform and classified crowd workers according to the experimental results. They summarized five types of crowdsourcing workers: proper worker, random worker, semi-random worker, uniform worker, and sloppy worker. It can be seen from the above literature that there is a growing need to effectively monitor the quality of task completion for crowdsourced annotation.

### 2.3. Human-in-the-Loop

Human-in-the-loop aims to integrate human knowledge and experience to train accurate predictive models at a low cost. It plays an increasingly important role in the machine learning pipeline stages, including data preparation, model training, and model validation. Ref. [69] summarized human-in-the-loop machine learning techniques, including data extraction, data integration, cleaning, labeling, iterative labeling, model training, and inference. Ref. [70] reviewed the current work on human-in-the-loop from a data perspective and classified them into three categories with a progressive relationship. As can be seen from the previous studies, to cope with the challenges brought by data labeling, many researchers build new datasets through human-in-the-loop data preparation techniques, thereby speeding up model iterations and reducing the cost of data labeling [18,71,72,73]. Ref. [71] applied a human-in-the-loop approach to improve the prediction results to determine the land cover change detection threshold. To improve the generalization performance of the deep learning model for change detection, the human-in-the-loop data preparation technology was applied to the preparation sample data for the deep learning remote sensing change detection, and the experts’ feedback was fed into the deep learning model. In the modeling process, human supervision and guidance were input to improve the quality of labeled sample data. It makes the model more intelligent in solving challenging and complex scene remote sensing change detection tasks and freeing people from the tedious image annotation work. This technique makes large-scale, specialized, and automated remote-sensing image annotation possible. 

### 2.4. Action Learning

Deep learning technology is widely used in remote sensing image interpretation [13,39,74,75]. Due to the variety of remote sensing image scenes, most detection models have problems such as weak generalization performance and poor adaptation to the background of the image area. The existing methods generally solve this problem by increasing the training sample size. However, in the actual change detection application scenario where a large amount of unlabeled image data can be obtained, it is not feasible to ask experts in the field of remote sensing to label massive image data. Labeling massive data with high quality has become one of the bottlenecks in training deep learning models and has attracted increased attention from researchers [76]. Active learning was first proposed by [77] used a method combining the weighted incremental dictionary learning method and the active learning idea for the hyperspectral image classification problem of deep learning. This method uses representative samples to train the model and get higher accuracy with a small amount of labeled data. Ref. [78] used active learning combined with contemporary deep network models in remote sensing image change detection. By selecting samples with high information content, the model can achieve the same performance by only labeling a small part of the samples.

## 3. Methods

### 3.1. Methods Overview

**Figure 1:**
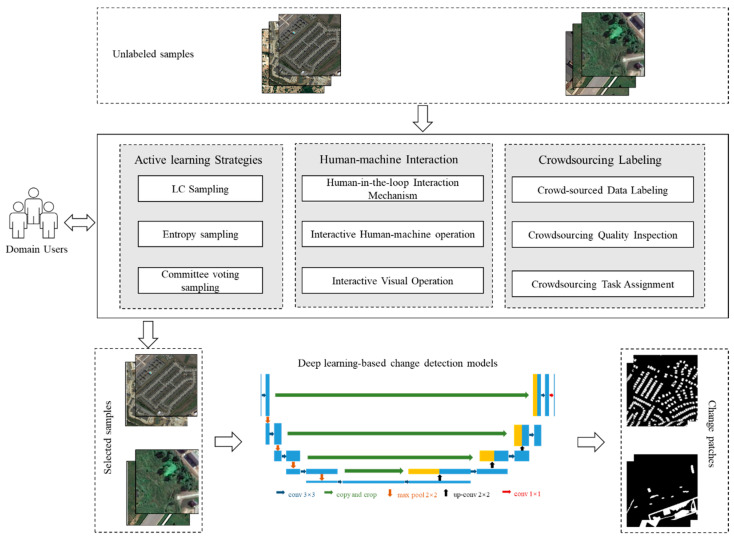
Framework Overview—Framework of automatic detection model integrating active learning, crowdsourcing, and human-in-the-loop techniques.

Currently, fully supervised learning is still the most compelling feature learning method in deep learning. The supervised learning algorithm trains a highly robust model depending on the scale and quality of the labeled samples. Most of the existing crowdsourced annotation research is biased toward image classification research, and there is very little research on providing annotated datasets for change detection. Moreover, no platform is integrated with the remote sensing change detection models with the computer interaction interface for crowdsourcing data labeling. Therefore, an efficient data annotation platform to obtain labeled sample data is needed, and the crowdsourced image labeling platform can be an effective solution. Due to the complexity of remote sensing image change scenes, the current automatic change detection methods still have problems, such as false alarms and missed detections, which cannot meet the needs of practical applications. The human-in-the-loop technology is proposed as a feasible solution to make the automatic detection models adapt to the changes of images with complex scenes by integrating experts’ experience into learning the change detection model and making the model more intelligent, relying on human intelligence. In this paper, the existing human-in-the-loop technique is used from the data perspective, and the model generalization can be enhanced by improving the quality of data annotation. The automatic change detection model and the crowdsourcing collaboration framework are shown in Figure 1.

This integrated change detection system comprises three key components. Firstly, active learning strategies are integrated into the human-in-the-loop paradigm to optimize the learning process’s efficiency and effectiveness, particularly during the training of machine learning models. The fusion of active learning techniques with human involvement creates an approach incorporating the strengths of both automated algorithms and human expertise. The second component focuses on human-machine interaction, specifically in the context of human-in-the-loop interaction. This interaction facilitates continuous quality control and assurance by empowering humans to review and validate data annotations, ensuring the model’s accuracy and reliability. The third and final component involves crowdsourcing labeling, a form of human-in-the-loop interaction. This approach entails outsourcing tasks, such as data labeling, to a large and diverse group of individuals. Crowdsourcing labeling offers advantages, including cost-effectiveness and the ability to handle large-scale projects efficiently. This component adds a valuable layer to the integrated system, leveraging the collective efforts of a diverse crowd to contribute to the data annotation process.

### 3.2. Human-in-the-Loop

**Figure 2:**
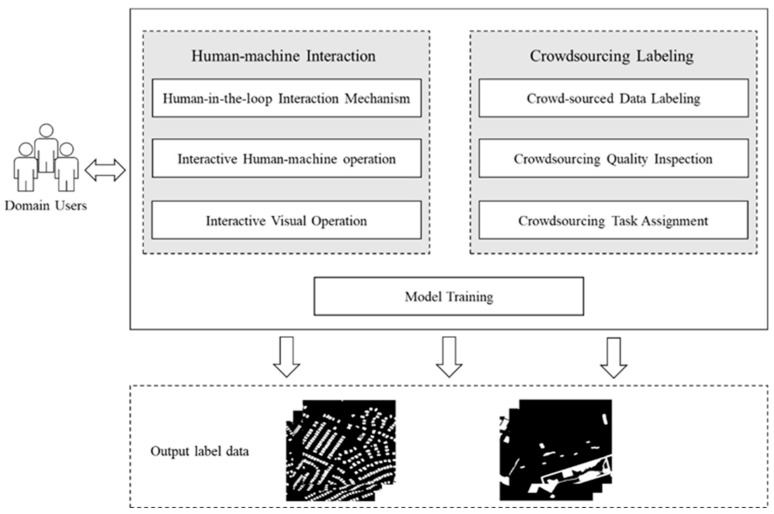
Human-in-the-loop flowchart.

To address the challenges brought by data labeling, many researchers build new datasets through human-in-the-loop data preparation techniques, thereby speeding up model iteration and reducing the cost of data labeling. Human-in-the-loop technology is applied to preparing remote sensing image interpretation data, mainly through the mutual iteration of manual labeling and algorithm model pre-labeling. Humans and humans perform review and correction, and humans and machines work together to apply the target model to the production of training sets to improve the efficiency of data preparation. It frees people from the tedious image annotation work, improves the accuracy and efficiency of model detection, and meets the needs of large-scale, specialized, and automated remote sensing image annotation. Moreover, the convenience of human-computer interaction is also the key to human-in-the-loop data preparation. The machine’s sample learning feedback can be obtained in time through a friendly visual operation interface. The human-in-the-loop machine learning data preparation is shown in Figure 2, which mainly consists of these three parts: human-computer interaction, crowdsourced labeling, and model training. In our proposed framework, human-in-the-loop technology plays an important role in addressing the challenges posed by data labeling, particularly in the context of remote sensing image interpretation for change detection. The application of human-in-the-loop technology involves a dynamic and iterative process, where manual labeling and algorithm model pre-labeling engage in mutual iteration. First, in the data labeling process, human annotators and algorithm models collaborate iteratively. The process involves manual labeling of remote sensing images, which is then complemented by pre-labeling by algorithm models. This mutual iteration allows for continuous refinement and improvement of the labeled data. The labeling data are then reviewed and corrected by humans, and this step ensures the quality and accuracy of the labeled information, addressing potential errors introduced during the pre-labeling phase. Humans and machines work collaboratively to apply the labeled data to the production of training sets. This collaborative effort enhances the efficiency of data preparation by leveraging the strengths of both human expertise and machine capabilities. This approach ensures the active involvement of humans in the labeling process, contributing to the accuracy, efficiency, and adaptability of change detection in remote-sensing images.

### 3.3. Crowdsourcing

**Figure 3:**
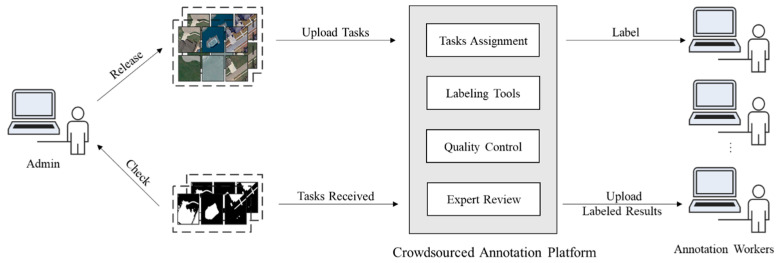
Crowdsourced remote sensing image change detection and annotation flow chart.

The general process of using crowdsourcing to label remote sensing image change detection datasets is shown in Figure 3. It mainly comprises annotation workers, crowdsourced annotation platforms, and administrators. This paper proposes a quality control model and an annotator qualification assessment model. The unqualified annotators were eliminated in time, and the result quality inspection was carried out on the results of these qualified annotators. The commonly used annotation quality evaluation algorithms, including the MV algorithm and EM algorithm, are used in the quality screening of image annotation results to improve the efficiency of remote sensing image change detection. 

#### 3.3.1. Annotator Qualification Assessment Model

The annotator assessment model is proposed to evaluate the ability of the annotator, which aims to categorize the crowd workers of their annotation qualifications, including (1) domain expert annotators, (2) highly-qualified annotators, (3) adequately-qualified annotators, and (4) unqualified annotators. This model excludes annotators who have malicious labeling behaviors. It assigns tasks to different qualifications of annotators based on the task difficulties predicted by the initial model prediction. The tasks that are difficult to interpret image changes will be assigned to crowd workers with high labeling qualifications. Tasks that are less difficult to interpret will be assigned to crowdsourcing workers with medium-level labeling qualifications (e.g., adequately qualified annotators), and the labeling results will then be handed over to domain experts for final quality inspection. The gold standard test task is used to detect the presence of malicious crowd workers in crowdsourcing quality control. These gold standard test tasks with known correct answers are added to the crowdsourced labeling task. According to the annotation results of examining the gold standard test task, the task completion quality of the crowdsourcing workers is evaluated, and the annotating qualifications and work attitudes of the crowdsourcing workers are evaluated. In the crowdsourced labeling qualification evaluation experiment in this work, to avoid the appearance of malicious labelers, the gold standard test tasks are hidden in the tasks distributed to the annotators so that the annotators are unaware of the existence of gold standard test tasks in advance. 

The annotator’s ability assessment algorithm uses an image similarity algorithm to compare the gold-standard task results annotated by the annotators with ground truth. In this work, the Euclidean distance algorithm is used to calculate the similarity between the results of the gold test data marked by the annotator and the ground truth. The Euclidean distance is widely used to measure the distance between two points in n-dimensional space [79]. Equation (1) is used to calculate the Euclidean distance.

(1) $$d(P_{im},P_{m})=\sqrt{\sum_{m=1}^{n}{\left(P_{im}-P_{m}\right)}^{2}}$$

where $P_{im}$ represents the *m*th pixel value in the image annotated by the annotator i, and $P_{m}$ represents the ground truth. The larger the calculated distance value *d*($P_{im},P_{m}$) is, the more significant the difference between the labeled result and the ground truth is. A bigger distance value indicates a smaller image similarity and a lower annotation quality. Therefore, the similarity *K* is calculated using Equation (2) below after obtaining the Euclidean distance.

(2) $$K=\frac{100}{1+d\left(P_{im},P_{m}\right)}$$

A threshold *K* is set to 0.35, and the annotators whose similarity value is lower than the threshold are judged as unqualified annotators, and their labeled results will be removed. As a result, the annotators above the threshold are judged as qualified.

#### 3.3.2. Annotation Quality Control Model

In the process of image annotation using crowdsourcing, the same annotation task needs to be distributed to multiple annotators, and then the results of these annotations are collected. After the unqualified workers are excluded by the annotator evaluation model, the quality inspection processing is performed on the collected marking results of qualified workers. Professional annotators check the quality and correct the annotation results of workers and finally obtain high-quality annotation results. Currently, among the crowdsourced annotation data result aggregation methods, the most used are the MV algorithm and the EM algorithm.

**Figure 4:**
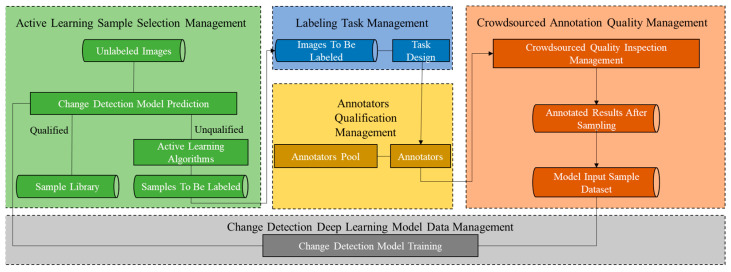
Flow chart of annotation quality control model.

Typical quality assurance methods are generally studied from the following two main aspects: (1) Crowdsourced annotator qualification evaluation: By mixing gold standard test data in the labeling task, the qualifications of the annotators are evaluated. The evaluation is mainly to check whether the labeling attitude of the annotator is serious and whether the ability meets the requirements and formulate a reasonable strategy for the later labeling task assignment. (2) Task results evaluation: Through the first step of qualification evaluation, remove the labeling results of unqualified labelers, and then combine the EM algorithm and MV algorithm to estimate the labeling quality accuracy of the results submitted by qualified labelers and filter out the labeling quality. Qualified results guarantee the quality of samples that are used for model training. The crowdsourcing quality control process is shown in Figure 4.

##### MV Algorithm

The MV algorithm is a highly versatile crowdsourcing quality control algorithm. This algorithm counts the votes for each tag in the results returned from the crowdsourcing task. The algorithm uses results from the majority of the annotators as the ground truth for the task. Studies have shown that the annotation quality accuracy obtained by the majority voting algorithm can even exceed that of a single domain expert [63]. As a benchmark algorithm for comparison experiments, the algorithm assumes that all the annotators have the same level of annotation. In the real world, each annotator’s attitude and professional background are different, so the credibility of the submitted labeling results is also different. This work integrates the annotation results of qualified annotators based on the annotation qualification ability of the annotators to avoid the interference of the annotation results of malicious annotators. The MV algorithm is applied to the quality assessment method of crowdsourced image segmentation, and those pixels of the same variable type labeled by at least half of the annotators are regarded as correct classification.

##### EM Algorithm

The EM algorithm performs a maximum likelihood estimation method on the accuracy of the labeled data of multiple annotators. The algorithm iterates sequentially until convergence and calculates the accuracy of each annotator’s annotation results to automate the assessment of the quality of the labeled images of the crowd workers. The EM algorithm is applied to the crowdsourced image segmentation quality assessment method, which is accomplished through the following two processes: (1) Using the annotations made by multiple annotators, the correct classification of each pixel is estimated. (2) By comparing the annotation submitted by the annotator with the estimated correct label classification, an overall annotation quality assessment of the results submitted by the annotator is obtained. The final output of the algorithm is the estimated correct answer for each annotation task and the overall error rate matrix for each annotator, which serves as a scalar value for each annotator’s annotation quality evaluation. Each crowdsourced labeling task is submitted to multiple annotators for labeling, and the quality of the labeling results is evaluated. The steps of the EM algorithm are shown in Algorithm 1.
**Algorithm 1** EM algorithm input: Given annotators’ label and error vector and priors for each pixel output: The probability that the annotator marks the pixel correctly priors = sum(segmentations)/len(segmentations) errors = self._m_step(segmentations,np.round(priors), segmentation_region_size, segmentations_sizes) Begin for _ in range(self.n_iter):  priors = self._e_step(segmentations, errors, priors)  errors = self._m_step(segmentations, priors, segmentation_region_size, segmentations_sizes) return priors > 0.5 End Return priors, errors

The detailed description steps are as follows:

Step 1: First, input an unknown actual classification label $T\left(O_{n}\right)$ of the crowdsourced image change labelling task, where the pixel value of the changed label is assigned a value of 1, and the pixel value of the unchanged label is assigned a value of 0;

Step 2: To measure the quality of the task completed by each annotator, the algorithm assigns the number of annotators (a) a matrix $M_{xy}^{a}$, which indicates that the actual classification of the crowdsourcing annotation task is the change class x, and the probability that annotator (k) assigns it to the unchanged class is y;

Step 3: The algorithm starts to execute the e-step, given the annotation results and error vectors of the annotators and the initial prior probability, use the Bayesian formula to calculate the posterior probability of each pixel classification category, and the algorithm continues to iterate until convergence (when the posterior probability is greater than 0.5, the algorithm stops iteration);

Step 4: Then execute the m-step, given the result of the annotator’s annotation and the posterior probability of each pixel finally obtained in the e-step, thereby estimating the annotation error probability vector of each annotator and obtaining the crowd worker’s annotation. The probability of correct change of class pixels, and finally, calculate the accuracy of a single image annotation.

### 3.4. Active Learning in Change Detection

**Figure 5:**
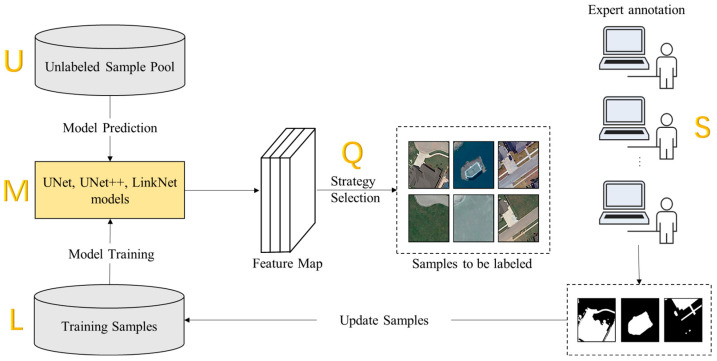
Flowchart of active learning in remote sensing change detection.

The purpose of active learning is to use as few labeled samples as possible to train the target model so that the model can focus on learning the most valuable samples as much as possible, and the model can also achieve high accuracy simultaneously. Since each sample has different contributions in deep learning model training, selecting difficult samples to classify helps improve the model’s generalization ability. To avoid the spatially redundant sample data of labeled samples, the active learning algorithm is introduced into the deep learning technology in remote sensing image change detection, which can alleviate the dependence on a large number of labeled samples in the process of model training, reduce redundant samples, and make full use of unlabeled samples. In remote sensing image interpretation, the main challenge of active learning is finding the most valuable samples from the massive unlabeled data. Active learning methods have two essential parts: a model learning module and a sample selection module. The active learning algorithm is an iterative process applied to deep learning remote sensing image change detection training, as shown in Figure 5.

The active learning process is mainly composed of five modules, as shown in Figure 5, including unlabeled data sample pool U, change detection deep learning algorithm models M, training sample set L, sample selection strategy Q, and remote sensing field expert annotation S. The specific steps are as follows: (1) Use the training samples to initialize the training change detection algorithm model to obtain the benchmark model, and then use the model to predict the unlabeled data and calculate the uncertainty score value of each sample; (2) select the appropriate sample selection strategy to obtain those samples that are difficult to classify and ask experts to annotate them; (3) add the annotated sample data to the training sample set and restart the model training; (4) stop iterative training if the model reaches the expected performance or the labeling budget, otherwise repeat the above (1), (2), (3) steps. Continuously update the training sample set to train the target model, and the model performance will gradually improve.

To apply active learning in the automatic detection model and the crowdsourcing collaboration framework, different sampling methods are used, including Least Confidence (LC), entropy sampling, and committee voting sampling. The sample selection method is the core of applying active learning in improving deep learning model performance. The comparative experiments were conducted, and the results are presented in Section 5.3.

#### 3.4.1. LC Sampling

The deep learning change detection model can score each pixel from the input image and classify it into a specific category based on the probability value. For example, in the binary remote sensing image change detection scenario, the model predicts two pixels in a sample as the changing class, and the probability values of the unchanged class are (0.52, 0.48) and (0.89, 0.11), respectively. The probability value of the first pixel judged by the model to be a change class is 0.52, and the probability value of the unchanged class is 0.48. The probability value of the second pixel judged by the model to be a change class is 0.89, and the probability value of the unchanged class is 0.11. It is more “difficult” for the model to distinguish the category of the first pixel, which indicates that this pixel is very close to the classification hyperplane, the category is easily wrongly judged by the model, and the confidence level is low. The mathematical equation of the least confidence sampling method is expressed as follows:

(3) $$S_{LC}^{*}={argmin}_{s}\left(P_{u}\left(\hat{y}|s\right)\right)$$

where $P_{u}\left(\hat{y}|s\right)={argmax}_{y}{(P}_{u}\left(y|s\right))$, $\hat{y}$ represents the maximum probability value of sample s in each class probability value predicted by the model, and u represents all parameter sets in the model training process. The algorithm idea of LC is to select the sample with the smallest corresponding probability value among the maximum class probability predicted by the model, that is, the sample with the least accurate model classification.

#### 3.4.2. Entropy Sampling

In statistics, the entropy value is often used to measure the stability of a system. A bigger entropy value indicates a weaker stability of the system, while a smaller entropy value indicates a more robust stability of the system. In deep learning model training, the cross-entropy function is usually used to calculate the loss value and evaluate the model’s performance. In this paper, the entropy value of the sample is calculated when the samples are predicted based on the change detection model. The samples with a relatively large entropy value are selected for annotation. The entropy method sampling is defined using Equation (4) below:

(4) $$S_{E}^{*}={argmax}_{s}-\sum_{i}P_{\theta }\left(y_{i}|s\right)\cdot lnP_{\theta }\left(y_{i}|s\right)$$

Compared with LC sampling, which only considers the probability value that the detection model for classifying the samples with the largest value, the entropy method sampling method considers the probability value of the detection model for all classes of the sample s.

#### 3.4.3. Committee Voting Sampling

The LC and entropy sampling methods only consider the uncertainty sampling results of a single model, and the selected samples are weak samples with uncertainty. The committee voting sampling considers the sample data that multiple models classify. When considering the prediction effects of all models, the entropy value can be used to measure the difficulty of the sample data being classified by these models. The most commonly used method in committee voting is the vote entropy method. This method selects the samples with high entropy values for prediction, which are the sample data from which these models are indistinguishable. The committee vote sampling is expressed mathematically as in Equation (5) below:

(5) $$S_{VE}^{*}={argmax}_{s}-\sum_{i}\frac{V(t_{i})}{M}\cdot ln\;\frac{V(t_{i})}{M}$$

where *M* represents the total number of model algorithms in the committee, $\sum_{i}V\left(t_{i}\right)=M$, and the parameter sets of each model are $\left\{q^{\;(1)},\text{…},q^{\;(M)}\right\}$. And these models are all trained by the same dataset, $t_{i}$ represents the sample *s* is classified as class i, and $V\left(t_{i}\right)$ indicates the number of models that classify the sample s as class i.

### 3.5. Design of the Crowdsourcing Change Detection Platform

**Figure 6:**
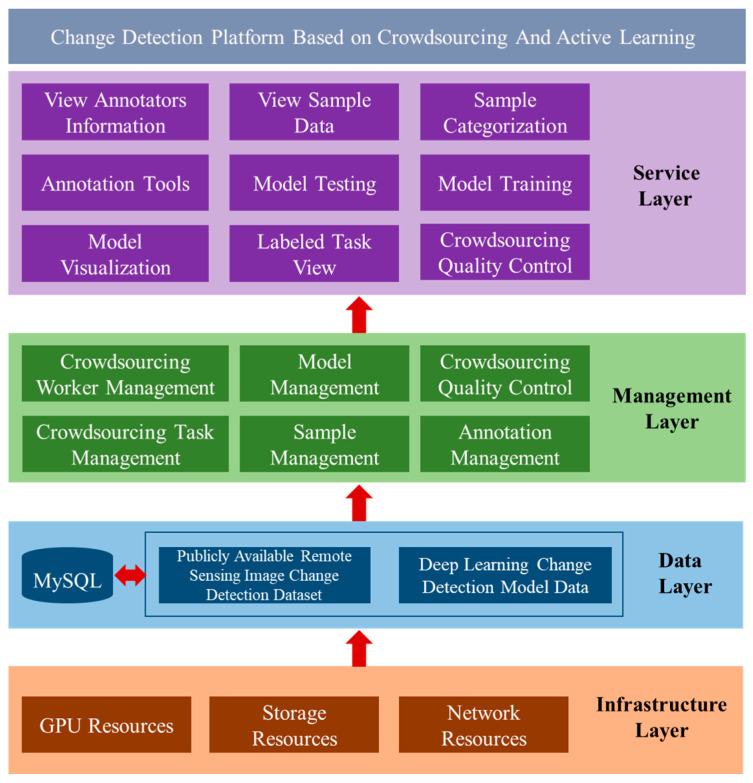
Architecture of crowdsourcing change detection platform.

The architecture design of the optical satellite remote sensing image change detection platform based on crowdsourcing is shown in Figure 6, which is divided into four parts: service layer, management layer, data layer, and infrastructure layer.

The service layer provides platform users with a friendly management interface for change detection model training. The main functions include crowdsourced worker information query, sample database query, sample data visualization, model database information query, visualizations on the training set and validation set, annotation task viewing, annotation task upload and download, model training, and testing.

The management layer is mainly for the managers of the crowdsourcing-based optical satellite remote sensing image change detection platform, mainly including crowdsourcing image annotation worker information management, public remote sensing image change detection sample library management, remote sensing image change detection deep learning model library management, platform login log management, crowdsourced labeling task management, and crowdsourced labeling quality control management.

A data layer is based on multisource public remote sensing image change detection datasets. Massive remote sensing image data and model data support the crowdsourcing-based optical satellite remote sensing image change detection platform. Also, it uses the file system storage method. Change detection sample library metadata and model metadata information are stored using the MySQL 8.0 database.

The infrastructure layer includes GPU computing resources required for change detection model training, storage resources for sample datasets, and network resources for data transmission. It provides physical resources for a crowdsourcing-based optical satellite remote sensing image change detection platform and change detection deep learning model training support.

Regarding the design of interfaces, the service layer is crucial. It facilitates user interactions and includes functionalities for data visualization, model training, and annotation task management. The design of these interfaces prioritizes user-friendliness, accessibility, and efficiency, allowing platform users to seamlessly perform tasks related to change detection model training and data annotation. The visualizations and query functionalities should provide a clear understanding of the annotated data, supporting effective decision-making during the model training process. Additionally, the annotation task management interface should enable easy upload and download of tasks, ensuring a smooth workflow for crowdsourced labeling. Annotated data is stored in databases; in this work, it is MySQL. Each annotation task is associated with a unique identifier, and the annotations are linked to specific data samples.

## 4. Data and Experiment

### 4.1. Experimental Dataset

**Figure 7:**
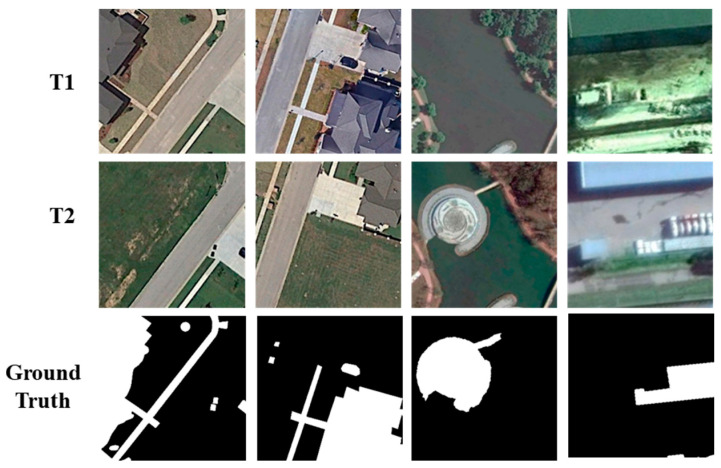
Example samples from the CDD dataset.

Two experimental datasets were used to validate the proposed crowdsourcing quality control and active learning models. The first experimental dataset used in this work is the CDD dataset released by Lebedev et al. [80] in 2018. The dataset records the fundamental, seasonal changes of remote sensing images. The dataset has a total of 16,000 images, the spatial resolution ranges from 3 to 100 cm, and the image size is 256 × 256 pixels, including 10,000 samples for the training set, 3000 samples for the test sets, and 3000 samples for the validation set. Variations in dataset annotations range from cars to large building structures (changes of unnatural objects) and natural objects such as from single trees to vast forest areas (natural changes of natural objects). Figure 7 shows some example images from the CDD dataset.

**Figure 8:**
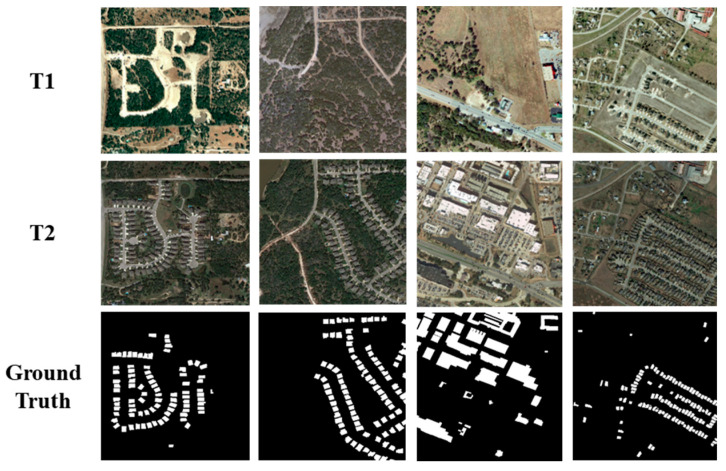
Example samples from the LEVIR-CD dataset.

The second dataset used in this work is the LEVIR-CD dataset published by Chen et al. [81] in 2020. The types of changes are mainly architectural changes, including changes from the soil, grass, and hardened ground to areas under construction or new buildings, and changes in buildings gradually disappear. The shooting areas of the dataset images are from 20 different places in Texas, USA, and the period is from 2002 to 2018 and contains a total of 31,333 building change patches. This dataset contains a total of 637 × 2 (pre-phase and post-phase) high-resolution remote sensing images with a resolution of 0.5 m. The image sizes are 1024 × 1024 pixels. There are 445 × 2 images in the training set, 64 × 2 images in the validation set, and 128 × 2 images in the test set. A sample LEVIR-CD dataset is shown in Figure 8.

### 4.2. Experiment Environments

The details of the experiment environments are presented in Table 1. 

### 4.3. Evaluation Metrics

*Accuracy*, *Precision*, *Recall*, and *F*1-*score* are used in the experiment to evaluate the model performance. The equations for calculating these evaluation metrics are shown in the Equations below.

(6) $$\mathrm{Accuracy}=\frac{\mathrm{TP}+\mathrm{TN}}{\mathrm{TP}+\mathrm{FP}+\mathrm{TN}+\mathrm{FN}}$$

(7) $$\mathrm{Precision}=\frac{\mathrm{TP}}{\mathrm{TP}+\mathrm{FP}}$$

(8) $$\mathrm{Recall}=\frac{\mathrm{TP}}{\mathrm{TP}+\mathrm{FN}}$$

(9) $$F1\text{-}\mathrm{score}=\frac{2(\mathrm{Precision}\times \mathrm{Recall})}{\mathrm{Precision}+\mathrm{Recall}}$$

To verify the situation where the ground truth is known, the evaluation index used in the experiment is used to measure the match between the final change detection region (CD) segmented by the EM algorithm, the MV quality assessment algorithm, and the ground truth change (GT). The most commonly used index evaluation method for image segmentation in most literature is MIoU, which stands out among many index evaluations due to its representativeness and simplicity and is the most commonly used accuracy evaluation method [82]. Considering the intersection area, I = CD∩GT of the marked change area, the real change area, and the union area U = CD∪GT, IoU = I/U. The formula for calculating the mean value of *MIoU* is shown in (3) below:

(10) $$\mathrm{MIoU}=\frac{1}{m+1}\sum_{x=0}^{m}\frac{S_{xx}}{\sum_{y=0}^{m}S_{xy}+\sum_{y=0}^{m}S_{yx}-S_{xx}}$$

where *m* refers to the change classes in total, $S_{xy}$ represents the number of pixels that belong to the class *x* but are predicted to be the class y and $S_{yx}$ represents class *y* but is predicted to be class x. The number of pixels with class changes, $S_{xx}$ represents the number of pixels that belong to class *x* and is predicted to be the class *x*.

### 4.4. Experiment Details

#### 4.4.1. Sample Selections Using Active Learning

In this section, random sampling is used as the benchmark. Comparative experiments were conducted for LC, entropy, and committee voting sampling based on deep learning change detection algorithms. Since the F1-score evaluation index takes both the precision and recall of the change detection model into consideration, the F1-score is used to measure the effectiveness of the active learning sampling method. The dataset used in the experiment is the CDD dataset (1000 training sets and 3000 test sets of actual seasonal change remote sensing images, and the image size is 256 × 256 pixels).

##### LC Sampling Experimental Steps

LC sampling experiment uses the CDD dataset and adopts U-Net [38] as the deep learning change detection model. The specific experiment steps of the LC sampling are as follows:

Step 1: Randomly select 6000 out of 13,000 samples from the training and test dataset to initialize the model;

Step 2: Predict the remaining 7000 samples and perform the softmax function operation on the linear output classification score of the last layer of the network model to map the predicted sample classification score into the probability value in the [0, 1] interval;

Step 3: Count the total number m of pixels, predicted values in [0.48, 0.52] in each sample, sort the probability values of these 7000 samples, and take the first 1000 samples close to 0.5 and integrate them into the previous training set. The model is then retrained;

Step 4: Repeat the above steps three times.

##### Entropy Sampling Experimental Steps

The entropy sampling experiment uses the CDD dataset and adopts U-Net as the deep learning change detection model. The specific experimental steps of entropy sampling are as follows:

Step 1: Randomly select 6000 out of 13,000 samples from the training and test dataset to initialize the model;

Step 2: Predict the remaining 7000 samples and perform the entropy formula operation on the output of the last layer of the network model to calculate the entropy value;

Step 3: Then, sort the entropy values calculated from the 7000 samples in descending order. Moreover, integrate the first 1000 samples into the previous training set and retrain the model;

Step 4: Repeat the above steps three times.

##### Committee Voting Sampling

The experiments use UNet [38], UNet++ [42], LinkNet [44], DeepLabV3plus [51], and FPN [49] change detection algorithm models, and the dataset uses the CDD.

The specific experimental steps of the committee voting sampling are as follows:

Step 1: Randomly select 6000 samples out of 13,000 samples from the training and test dataset to initialize the model;

Step 2: Use UNet, UNet++, LinkNet, DeepLabV3plus, and FPN models to predict the remaining 7000 samples. During the prediction process, the entropy formula operation is performed on the output of the last layer of the network model to calculate the entropy value of each sample;

Step 3: Then, take the first 1000 samples with large entropy predicted by these five models, integrate them into the previous training set, and retrain the model;

Step 4: Repeat the above steps.

#### 4.4.2. Crowdsourcing Annotator Ability Assessment Experiment and Annotation Quality Assessment Experiments

It is not easy to evaluate the annotators’ labeling work, attitudes, and qualifications reasonably and objectively. To conduct experiments more realistically, a certain amount of gold standard test data are mixed with the annotation tasks distributed to the annotators. The experiment selected a part of the images in the LEVIR-CD dataset as the annotation tasks and randomly selected half of the images as the gold standard test.

For the annotation experiments, 20 college students were recruited to participate in the annotation quality test of this task. Ethics permission was obtained from the School of Computer and Information Technology at Northeast Petroleum University Research Ethics Committee. Written informed consent was obtained from each participant before they enrolled in the annotation task. Among the 20 college students, 10 were experts (professional group) who were familiar with remote sensing images and had done relevant annotating work. The other 10 had no prior experience with remote sensing images, and it was their first-time completing image annotation tasks (non-professional group). The experimental dataset comprised 14 pairs of images randomly selected from the training set of LEVIR-CD, and 50% (seven pairs of images) of the images were randomly selected as the gold standard test data. The objects marked in the experiment are mainly the change of the house, and there are about 1800 house change patches in this experimental task, and there are about 1000 house change patches in the randomly selected gold standard task for the test. The annotators did not know the existence of seven pairs of gold standard test data in advance.

Annotation quality assessment experiments are done by comparing different algorithms, including MV and EM algorithms. The objects selected for the annotation quality assessment are from the annotations made by the selected qualified annotators.

## 5. Results

### 5.1. Crowdsourcing Annotator Ability Assessment Experiment Results

**Figure 9:**
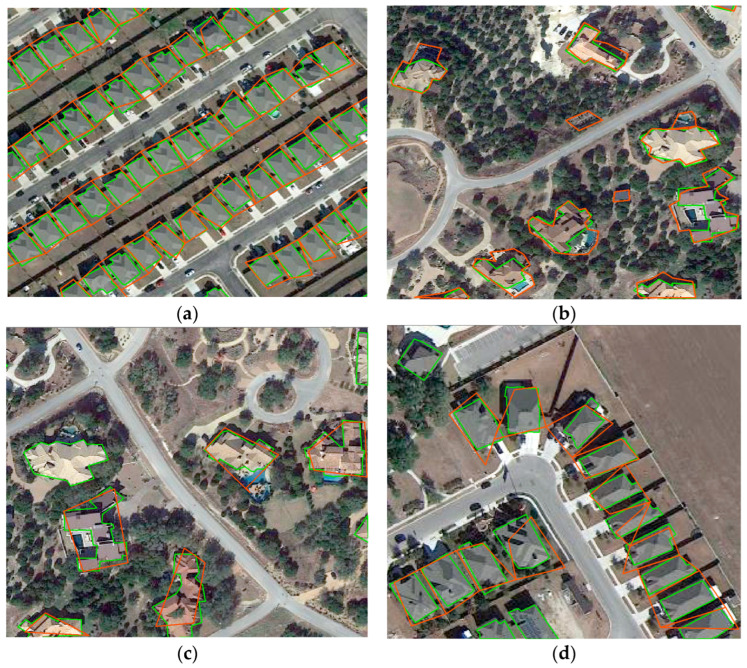
Screenshots of the annotation results of two unqualified annotators, (**a**,**b**) were annotated by the same annotator, (**c**,**d**) were annotated by another annotator, the red outline is the annotator’s annotation, and the green outline is the ground truth.

The results of the annotator ability assessment experiment are presented in Table 2. Among the 20 people in the experiment, there are two people whose average threshold K of seven images is lower than 0.35, which is 10% of the total number of annotators. By observing the labeling results from these two annotators, as shown in Figure 9, there are two main reasons for the inaccuracy. The first reason is that the attitudes of the annotators are not serious when annotating. It can be seen from Figure 9a that when the annotators label dense rows of houses, the labeling is relatively rough, and the gap area between the houses is also marked as part of the changed target. The second reason is that the annotator has errors and omissions during annotation due to the lack of professional background in remote sensing image interpretation. It was found that the two unqualified labels belong to the second group of non-professionals. It can also be seen from Figure 9b–d that because these two annotators are unfamiliar with the field of remote sensing and lack the relevant background knowledge, there are many misjudgments in judging the change of the house. The misjudgments include marking the pool and house shadow and weeds near the house as a house. 

Through the analysis of the similarity threshold K of the above experimental results, 20 people are categorized according to the similarity threshold K, as shown in Table 3. There are six categories: the annotators whose average threshold K is greater than 0.5 are professional annotators. Annotators with a threshold K ∈ (0.475, 0.5] are excellent annotators, those with a threshold K ∈ (0.425, 0.475] are good annotators, and a threshold K ∈ (0.4, 0.425] are adequate annotators. Annotators with the threshold K ∈ (0.35, 0.4] are average annotators, and when the threshold K is less than 0.35, they are unqualified. Categorizing the annotation qualifications of crowdsourcing workers is helpful for crowdsourcing task allocation and further, improving the quality of the annotation results.

### 5.2. Experiment Results of Annotation Quality Assessment

The experimental results of the annotation ability assessment of the annotators are shown in Table 2. Among the 20 annotations, 18 qualified and 2 failed. The labeling results of these two unqualified annotators are removed. This section applies the MV and EM algorithms for the quality inspection of the labelling results of these 18 qualified annotators. Each algorithm will be applied to six groups of experiments. We randomly select 3, 6, 9, 12, 15, and 18 annotators and use their annotation results as the first, second, third, fourth, fifth, and sixth groups of experiments.

**Figure 10:**
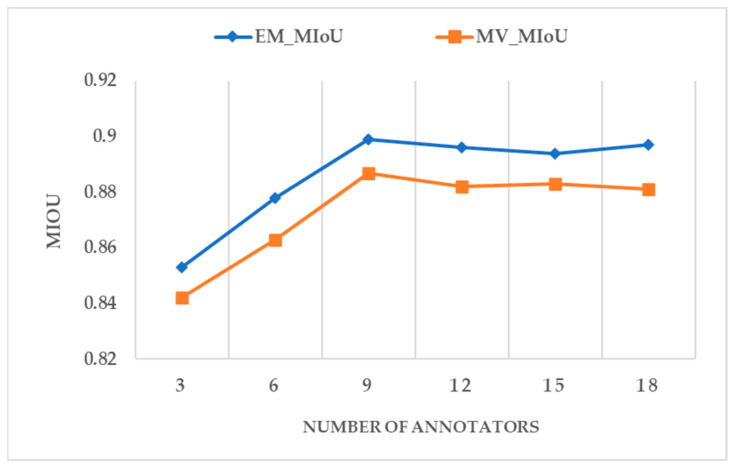
The accuracy of the answers is estimated by the MV algorithm and the EM algorithm under the real answer.

**Figure 11:**
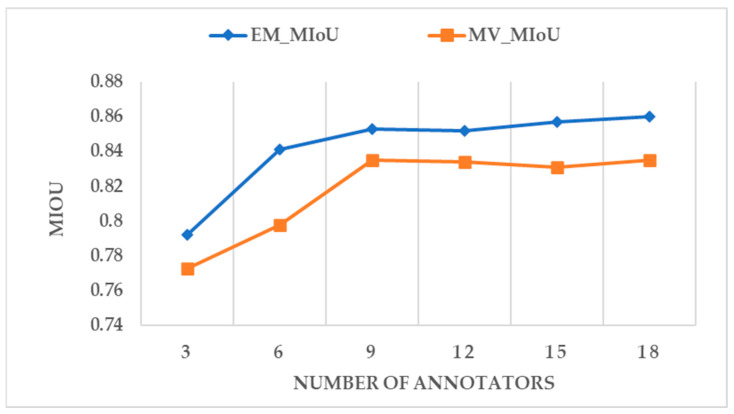
The accuracy of the annotator under the MV algorithm and the EM algorithm to estimate the answer.

Figure 10 shows the accuracy of the answers estimated by the MV algorithm and the EM algorithm where the ground truth is known. Figure 11 shows the accuracy of the annotation results of the annotators using the MV algorithm and the EM algorithm, respectively. The accuracy of the algorithm’s estimated answer is consistent with the accuracy of the standard answer. The EM algorithm is more reliable than the MV algorithm in measuring the results of the annotators. The MIoU values of the EM and MV algorithms vary with the number of annotators. The overall performance of the EM algorithm is slightly higher than the MIoU accuracy index of the MV algorithm. For the MV algorithm, the weighting for each annotator is set the same, which does not consider each annotator’s different attitudes and image interpretation abilities. The results verify that the results agreed by most people are not necessarily the actual results. There is some correlation between the number of annotators participating in the image change annotation task and the MIoU value. It can be seen from the six sets of experimental results that nine annotators completed the results that achieved the highest accuracy. Due to the limitation of the number of annotators in the experiment, there is currently no understanding of the changes in the average annotation accuracy when more annotators with different professional backgrounds participate in the annotation experiment. It can be seen from the current experimental results that the selection of an appropriate number may be related to the quality and accuracy of the annotation results. The EM algorithm is more suitable for measuring the quality of the annotation results of the annotators than the MV algorithm.

### 5.3. Experiment Results of Active Learning Sample Selection

**Figure 12:**
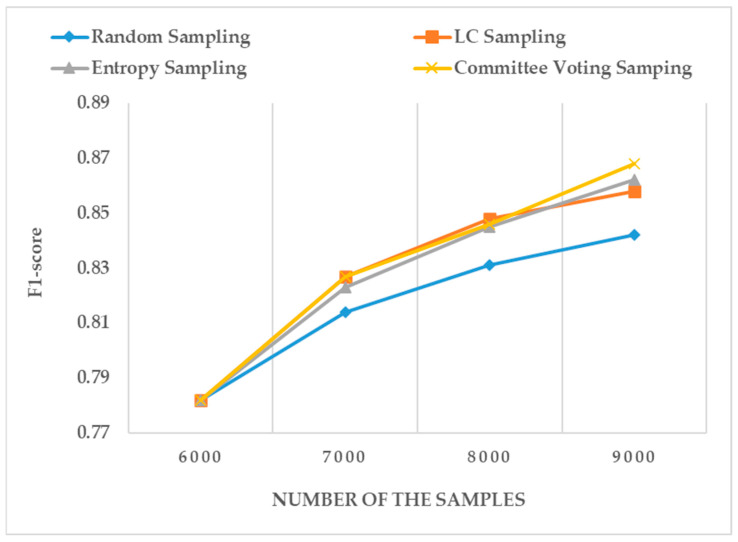
Example samples from CDD dataset.

Different sampling techniques were used in selecting appropriate samples for change detection models. As shown in Figure 12, with the increase in the dataset, the committee voting sampling method is the best among the three methods based on active learning. Random sampling only calculates the uncertain sampling results of a single model. The committee voting sampling method makes the prediction based on different detection models and selects the sample with the highest consistency entropy value for labeling. From the experimental results, under the same detection accuracy F1-score value, the LC sampling, entropy sampling, and committee voting sampling methods require fewer training samples than the random sampling method. It thoroughly verifies that the active learning method should be introduced into deep learning-based change detection without any labeled data. Only a small number of samples need to be labeled using the active learning method, and the ideal change detection accuracy can be achieved. The results show that active learning can reduce labeling costs and improve change detection efficiency.

### 5.4. Online Platform

**Figure 13:**
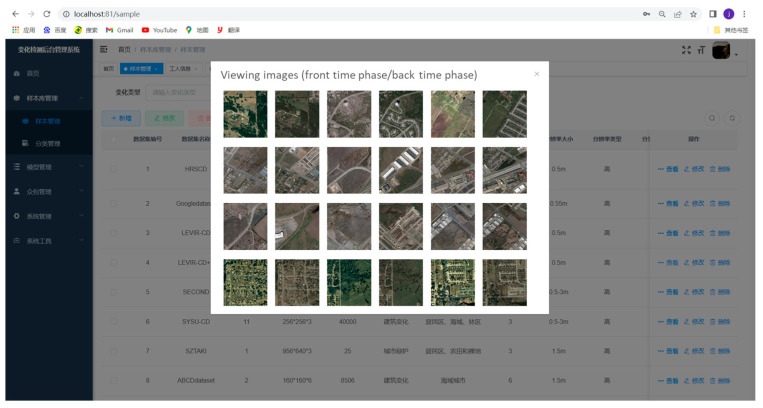
The visualization interface of the developed platform.

**Figure 14:**
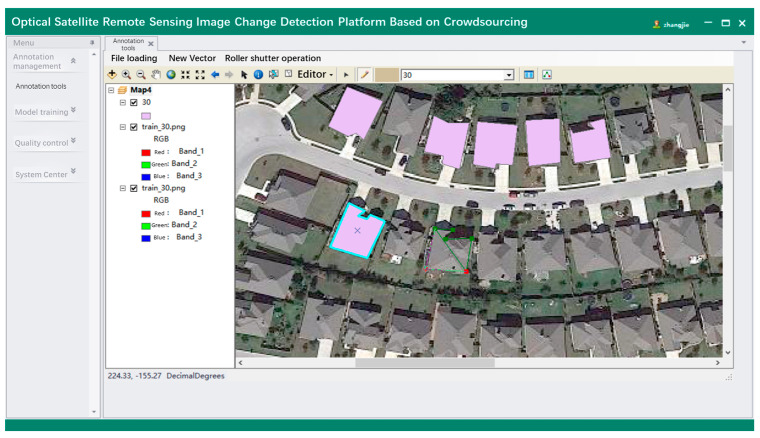
The annotation interface for change detection.

**Figure 15:**
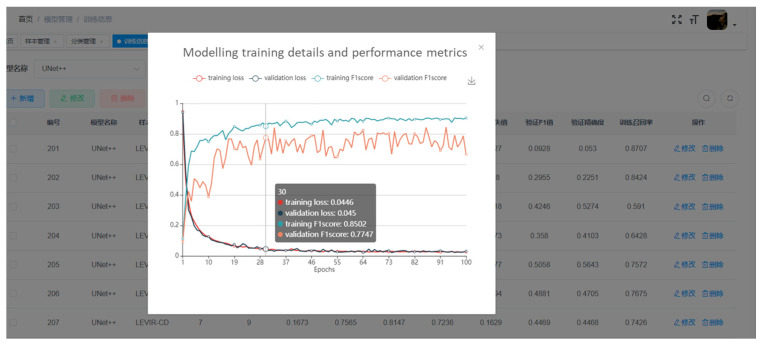
The interface for modeling training details and performance metrics.

The development of this remote sensing online platform for change detection based on crowdsourcing consists of the following stages: data sample management, annotation tools realization, and change detection model management. To reduce the problem of overfitting and insufficient generalization ability of the change detection model, a large number of high-quality samples with diverse change categories are required for training the change detection model. Managing diverse remote sensing image change detection sample libraries is the data basis to support the intelligent change detection application of the platform. Figure 13 shows the visualization of the interface for different phases of the change detection images. In remote sensing image change detection, reducing the time and cost of annotating samples is critical. The easy use of labeling tools is one of the solutions to improve labeling efficiency and user experience, which further supports providing samples for training deep learning-based change detection models. The function module designed within the labeling tool mainly includes raster data loading, creating a vector shapefile and displaying it, and providing basic map operations and polygonal surface labelling functions. Figure 14 shows the annotation interface for change detection. Furthermore, the platform provides visualization for modeling training details and performance metrics. The interface for training loss visualization is shown in Figure 15.

## 6. Conclusions

This paper proposes a remote sensing change detection framework that integrates crowdsourcing, active learning, and human-in-the-loop techniques to improve the generalization of the change detection model and adaptability to the background of the change detection regions by improving the quantity and quality of label data. The platform transforms the manual interactive visual interpretation method into a human-machine collaborative intelligent interpretation. Automatic detection and crowdsourcing collaborative process models are constructed under different application scenarios in remote sensing image change detection. A technical framework of automatic detection model and crowdsourcing collaboration is proposed. The low-cost and high-efficiency crowdsourcing model is used to solve the problem of weak model generalization ability caused by the shortage of sample data in new scenarios. The human-in-the-loop technology and active learning method are introduced to effectively use prior human knowledge and further reduce the cost of data annotation. A quality control model for crowdsourced image change detection is built with consideration of the annotator’s qualification and ability. The generation of noise samples is mainly controlled from two aspects. First, use the gold standard task test data to classify and evaluate the annotation qualifications of the annotators and eliminate the annotators with unqualified annotation qualifications. The EM algorithm and the MV algorithm filter the quality of the annotation results and filter out the samples with unqualified quality to ensure the accuracy of the quality of crowdsourced image annotation. Active learning has been used to reduce the use of the large, labeled dataset, and different data sampling methods were compared. The results show that the committee voting sampling method is the best among the three methods.

## Figures and Tables

**Table 1:** Experimental environments.

Experiment Environments	Details
GPU	NVIDIA GeForce RTX 2080Ti GPU (11264M)
CPU	Intel i9-9900KF CPU (3.60 GHz)
CUDA	10.2
RAM	32 GB
Operating system	Ubantu18.04
Development framework	Python 3.7, PyTorch 1.5.1

**Table 2:** Annotator ability assessment experiment results.

Annotator ID	T1	T2	T3	T4	T5	T6	T7	Average K Value
0001	0.365	0.403	0.414	0.426	0.368	0.396	0.359	0.390
0002	0.444	0.461	0.428	0.436	0.368	0.387	0.367	0.413
0003	0.486	0.448	0.436	0.454	0.441	0.413	0.387	0.438
0004	0.483	0.369	0.397	0.411	0.377	0.423	0.409	0.410
0005	0.582	0.451	0.468	0.465	0.475	0.417	0.448	0.472
0006	0.511	0.483	0.444	0.487	0.441	0.431	0.402	0.457
0007	0.456	0.391	0.437	0.381	0.362	0.371	0.369	0.378
0008	0.410	0.363	0.328	0.369	0.302	0.327	0.280	0.340
0009	0.387	0.346	0.373	0.383	0.344	0.360	0.304	0.357
0010	0.549	0.487	0.474	0.556	0.481	0.443	0.426	0.488
0011	0.465	0.466	0.455	0.397	0.416	0.397	0.396	0.427
0012	0.512	0.458	0.448	0.479	0.427	0.410	0.408	0.449
0013	0.538	0.508	0.469	0.512	0.472	0.426	0.434	0.480
0014	0.508	0.477	0.459	0.503	0.432	0.431	0.407	0.459
0015	0.407	0.349	0.360	0.354	0.339	0.416	0.359	0.369
0016	0.422	0.361	0.330	0.362	0.358	0.350	0.317	0.357
0017	0.493	0.492	0.540	0.507	0.527	0.505	0.480	0.506
0018	0.371	0.338	0.344	0.375	0.310	0.307	0.279	0.332
0019	0.441	0.418	0.451	0.408	0.353	0.393	0.369	0.405
0020	0.395	0.390	0.375	0.391	0.383	0.361	0.335	0.376

**Table 3:** Annotator’s annotation qualification evaluation classification description table.

Qualification Numbering	Annotator Qualification Description (K Is the Similarity Threshold)
1	Professional annotator, K > 0.5
2	Excellent annotator, 0.475 < K < 0.5
3	Good annotator, 0.425 < K < 0.475
4	Adequate annotator, 0.4 < K < 0.425
5	Average annotator, 0.35 < K < 0.4
6	Unqualified annotator, K < 0.35

## Data Availability

The data that support the findings of this study are available from the author, ZBW, upon reasonable request.
